# Using personalized 3D printed Titanium sleeve-prosthetic composite for reconstruction of severe segmental bone loss of proximal femur in revision total hip arthroplasty: A case report

**DOI:** 10.1097/MD.0000000000018784

**Published:** 2020-01-17

**Authors:** Xingshan Wang, Hui Xu, Ji Zhang

**Affiliations:** Department of Orthopedic Surgery, Beijing Jishuitan Hospital, 4th clinical medical college of Peking University, Xicheng District Xinjiekou, Beijing, PR China.

**Keywords:** 3D printing, bone loss, reconstruction, revision total hip arthroplasty

## Abstract

**Rationale:**

Allograft-prosthetic composites (APCs) and proximal femoral replacement have been applied for reconstruction of severe segmental femoral bone loss in revision total hip arthroplasty. The outcomes are encouraging but the complication rate is relatively high. Considering the high complication rates and mixed results of APCs and megaprosthesis, we presented a case using personalized 3D printed Titanium sleeve-prosthetic composite for reconstruction of segmental bone defect.

**Patient concerns:**

A 73-year-old woman presented to the emergency department on account of acute severe pain of the left hip without history of trauma. She had undergone a cemented total hip arthroplasty for osteonecrosis of femoral head at the left side in 2000. In 2013 she underwent a cemented revision total hip arthroplasty as a result of aseptic loosening of hip prosthesis. She denied obvious discomfort prior to this episode since the revision surgery in 2013.

**Diagnosis:**

According to the clinical history, imaging and physical examination, we confirmed the diagnosis of severe segmental bone loss of proximal femur and fracture of prosthetic stem. The femoral bone defect was evaluated using the Paprosky classification system and rated as Type 3B, and the acetabular bone defect was rated as Type 2C.

**Interventions:**

In this study, we present the first case of severe segmental bone loss of proximal femur in revision total hip arthroplasty that was successfully treated using personalized 3D printed Titanium sleeve-prosthetic composite

**Outcomes:**

At the 2-year follow-up, the patient was symptom free with a Harris Hip Score of 91. Radiographs showed excellent osteointegration between the interface of sleeve-prosthetic composite and the host bone, with no signs of implant loosening or subsidence.

**Lessons:**

Despite the absence of long term results of 3D printed Titanium sleeve-prosthetic composite reconstruction, the good clinical and radiological outcome at 2 years follow up implied its potential role for reconstruction of segmental femoral bone defect in revision THA.

## 1 Introduction

Total hip arthroplasty (THA) has gained great success in the past several decades. More than one million total hip replacements are done worldwide each year.^[1]^ Despite of the high survival rates of THA, the number of revision THA continues to increase due to aseptic loosening, dislocation, infection, and other reasons. Bone stock deficiency is a common challenge in revision THA and it is often exacerbated during implant removal. Reconstruction options vary according to the remaining bone quality. At the femoral side, allograft-prosthetic composites (APCs) and proximal femoral replacement is the preferred choice for severe segmental bone loss.^[2–5]^ The results of these procedures is encouraging but the complication rate is relatively high.^[6]^ Disease transmission, infection, graft absorption, aseptic loosening, or abductors deficiency, often compromises the results.^[3,4,7–9]^ To avoid the possible complications of APCs, we presented a case of revision THA using personalized 3D printed sleeve augment-prosthetic composite to manage severe bone loss and discontinuity of proximal femur secondary to osteolysis and fatigue fracture of femoral component.

## 2 Case presentation

### 2.1 Ethical statement

The procedures performed in this study were in accordance with the ethical standards of the ethical committee in our hospital. Informed consent for publication has been obtained from the patient.

### 2.2 Clinical data

**Figure 1:**
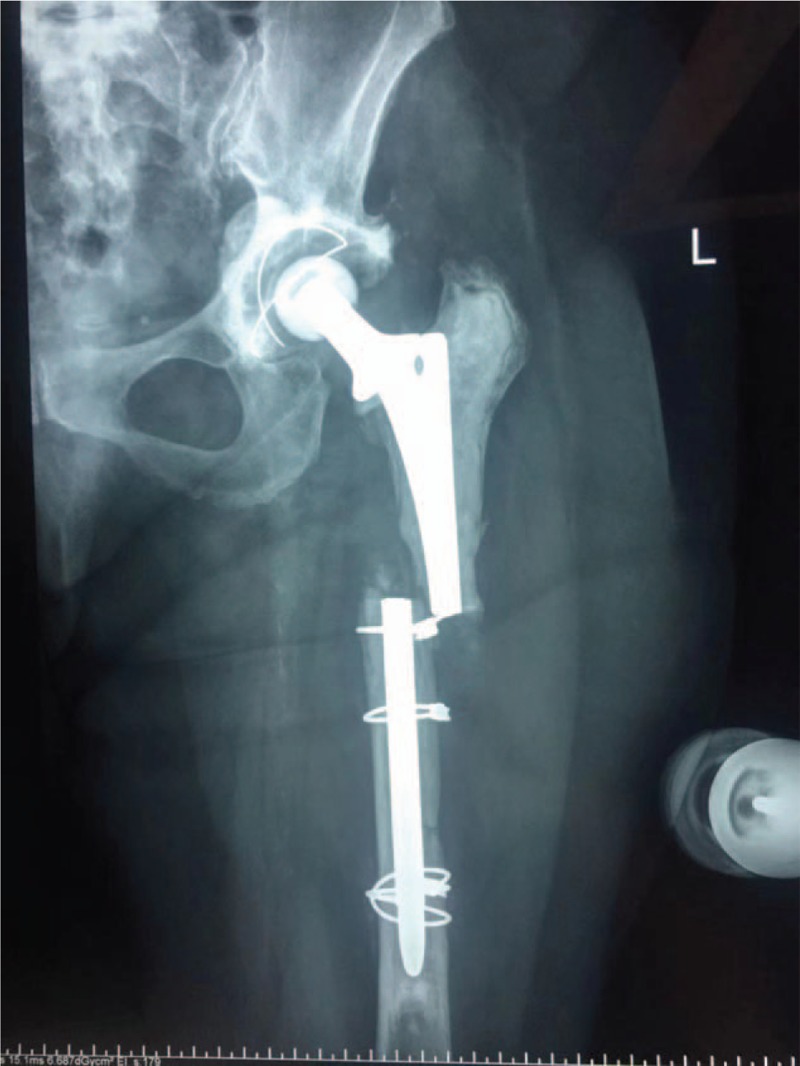
Radiograph demonstrated severe segmental bone loss of proximal femur and fracture of prosthetic stem. The femoral bone defect was evaluated carefully using the Paprosky classification system and rated as Type 3B, and the acetabular bone defect was rated as Type 2C.

A 73-year-old woman presented to the emergency department on account of acute severe pain of the left hip without history of trauma. She had undergone a cemented total hip arthroplasty for osteonecrosis of femoral head at the left side in 2000. In 2013 she underwent a cemented revision total hip arthroplasty as a result of aseptic loosening of hip prosthesis. She denied obvious discomfort prior to this episode since the revision surgery in 2013. On physical examination, tenderness, swelling and deformity of left thigh was noted. Radiographs demonstrated severe segmental bone loss of proximal femur and fracture of prosthetic stem. The femoral bone defect was evaluated carefully using the Paprosky classification system^[10]^ and rated as Type 3B, and the acetabular bone defect was rated as Type 2C (Fig. 1). All of the preoperative laboratory investigations (white blood-cell count, C-reactive protein level, and erythrocyte sedimentation rate) were within normal limits.

### 2.3 Preoperative preparation

**Figure 2:**
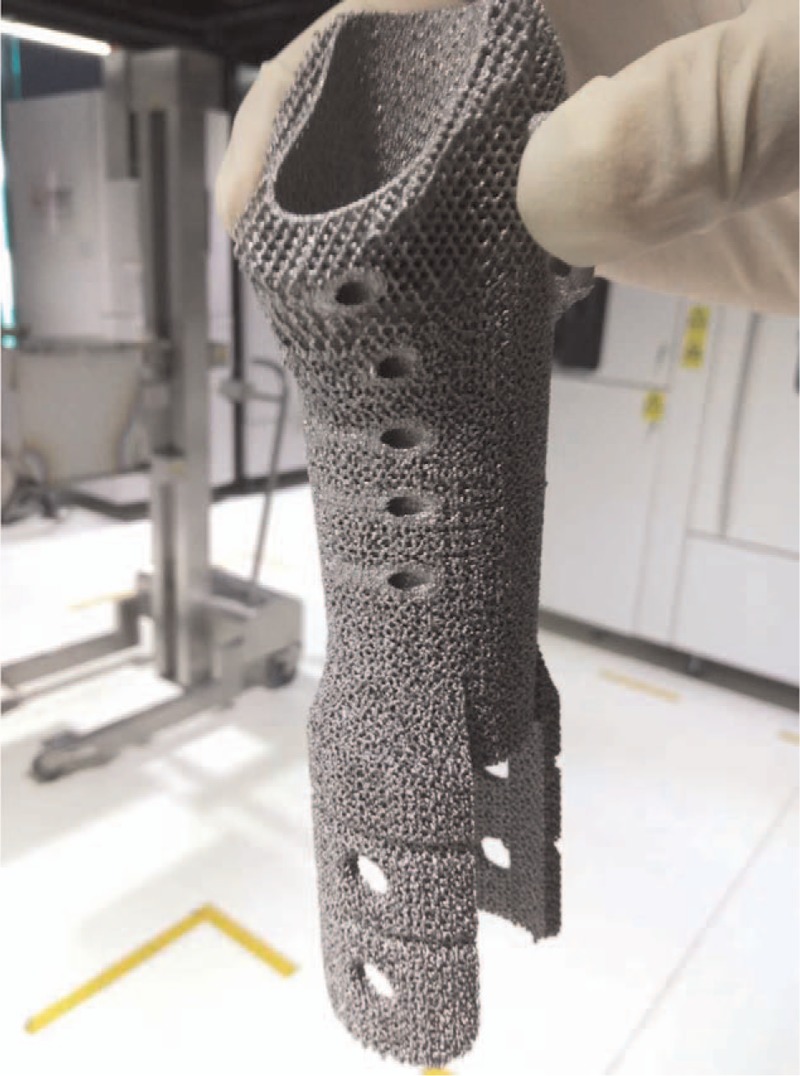
The photo showed a personalized 3D printed Titanium porous sleeve augment to manage the proximal femoral bone defect. The sleeve was customed using electron beam melting (EBM) 3D printing technology, projected to gain intimate contact with the inner bone surface of the greater trochanter. Two distal wings of the sleeve were designed to hold the cortex surface of residual isthmus.

**Figure 3:**
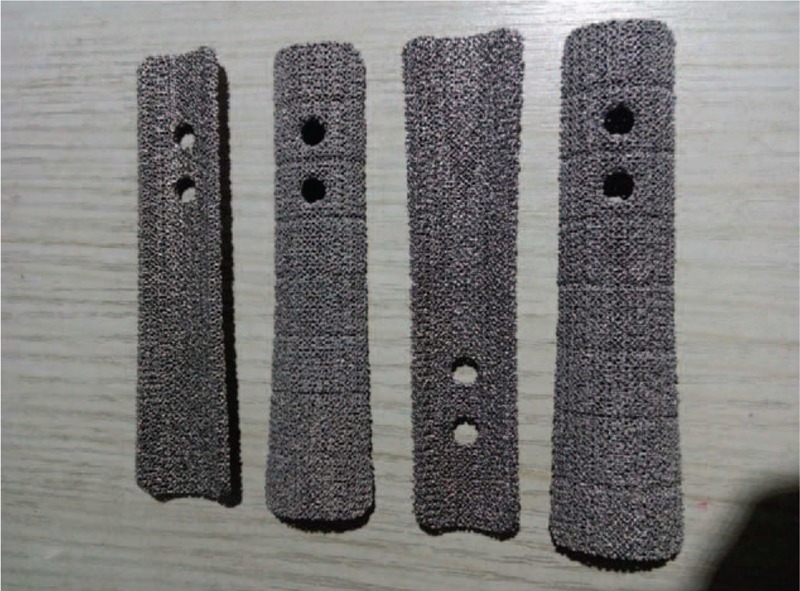
The photo showed porous coated Titanium plate augments was designed to bridge the defect between proximal femur and residual isthmus cortex, and be fixed by screws and cables.

We reviewed previously reported studies aimed for revision THA, in which, allograft-prosthetic composites and proximal femoral replacement was preferred to reconstruct large segmental femoral bone deficiencies. Considering the high complication rates and mixed results of APCs and megaprosthesis, we decided to use a personalized 3D printed Titanium porous sleeve augment combined with a modular tapered stem to manage the proximal femoral bone defect. The sleeve was customed using electron beam melting (EBM) 3D printing technology,^[11]^ according to computed tomography scan and 3D model data, to achieve intimate contact with the inner bone surface of the greater trochanter. Two distal wings of the sleeve were designed to hold the cortex surface of residual isthmus (Fig. 2). A porous coated Titanium plate was designed to bridge the defect between proximal femur and residual isthmus cortex and be fixed by screws and cables (Fig. 3). The shape and size of sleeve were designed according to radiological measurements prior to surgery to produce the personalized implant. The 3D structure of sleeve and Titanium plate were designed using computer assisted design (CAD) software (Magics), and the data were stored in STL file format. The porous architecture was designed based on a dodecahedron unit cell with a pore size of about 600 μm, strut diameter of about 500 μm and porosity of about 70% (Fig. 2). This architecture was adopted because it demonstrated that the pore size at this range is beneficial for in-growth of bone and vessels.^[12]^ Then the implants were prototyped using EBM Q10 system (Acram AB, Sweden). The porous structure on the surface of implants was constructed to facilitate bone ingrowth, and imitate the biomechanic properties of the host bone. Consent was obtained from the patient with full disclosure on the potential benefits and risks.

### 2.4 Operative procedures

**Figure 4:**
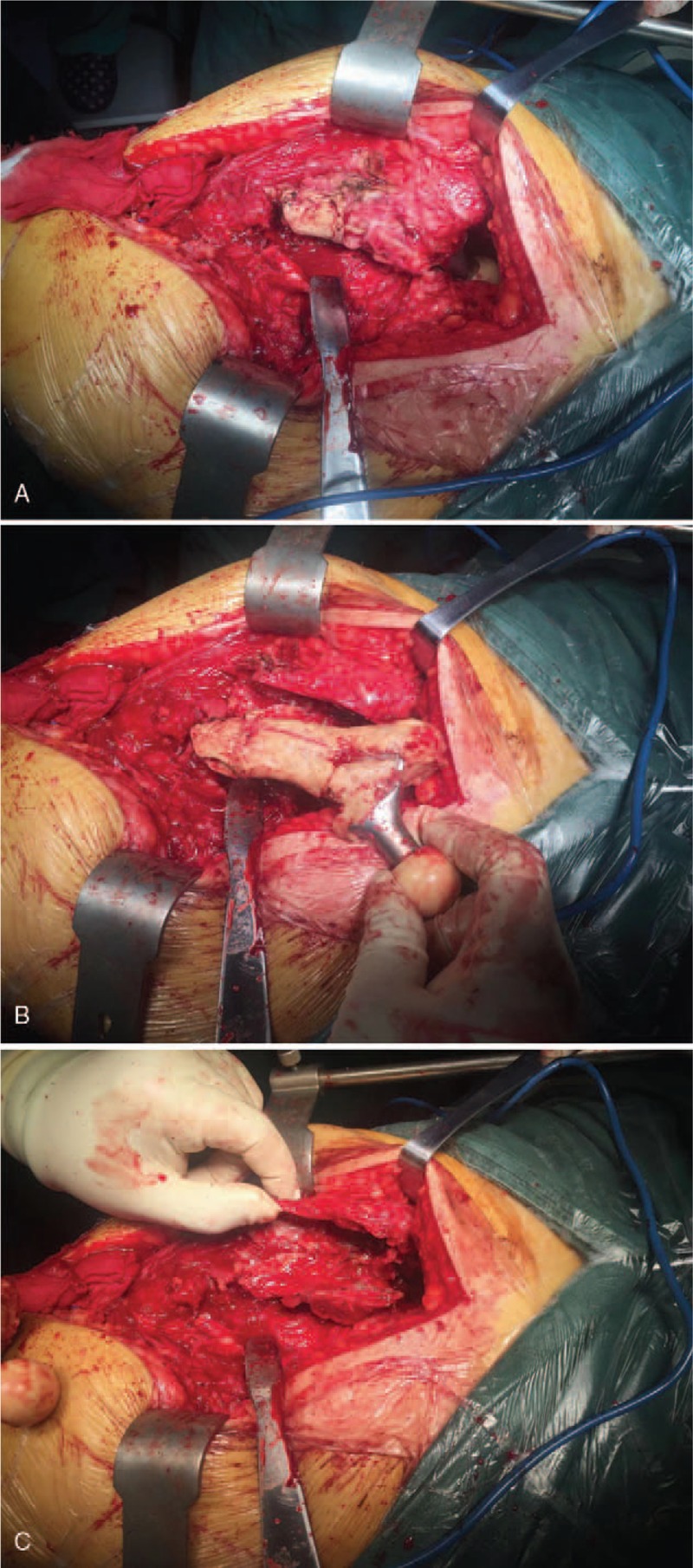
Intraoperative photo (A) showed an extended posterior approach was made through previous posterolateral incision. The bone stock adjacent to the fractured site was compromised and the surrounding cortex was absent secondary to osteolysis and the previous revision procedure. As shown in photo (B), extended trochanteric osteotomy was performed to facilitate the removal of the femoral component and cement. Photo (C) showed the severe bone loss of proximal femur after removal of the implants.

**Figure 5:**
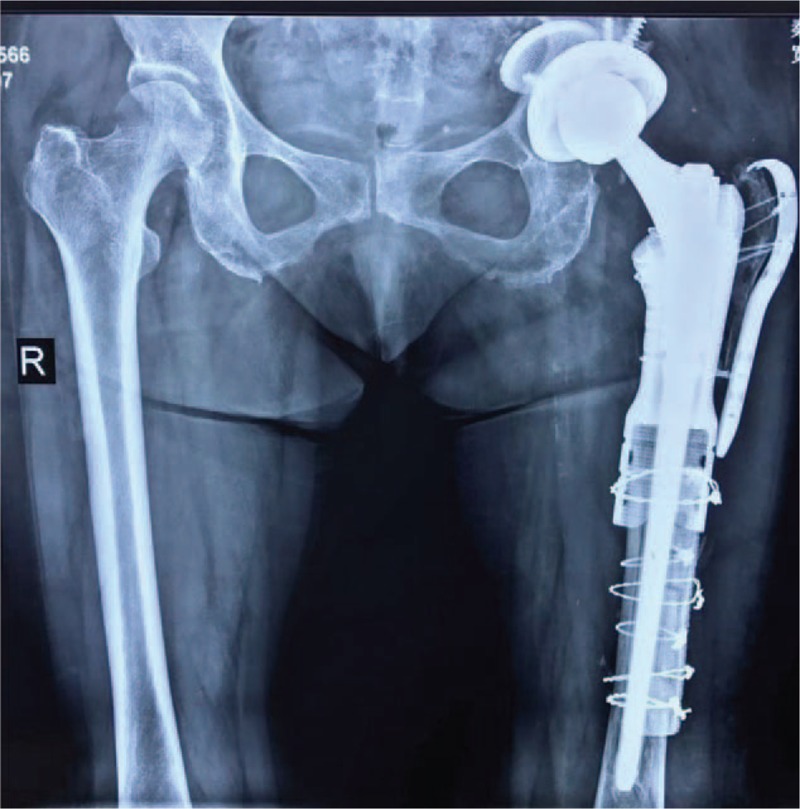
Postoperative radiograph demonstrated good restoration of hip center and reconstruction of proximal femur after surgery.

The surgery was performed as planned. An extended posterior approach was made through previous posterolateral incision. The location of prosthetic fracture site was 6.5 cm below the lesser trochanter. Both the proximal and distal part of the fractured stem was cemented well with massive cement filled in the femoral canal. The bone stock adjacent to the fractured site was compromised and the surrounding cortex was absent secondary to osteolysis and the previous revision procedure. The length of the bone defect is about 8 cm. Extended trochanteric osteotomy (ETO) was performed to facilitate the removal of the femoral component and cement (Fig. 4). The acetabular component was removed without difficulty. The acetabular bone defect was reconstructed with a 60 mm porous Titanium cup and 1 Titanium augment. A modular tapered stem was inserted into the personalized Titanium sleeve, and then the composite was implanted into the femoral canal. The two distal wings of the sleeve held the cortex of residual isthmus as planned. The stem and the sleeve was combined by cement. The greater trochanter was attached to the porous surface of the sleeve, and the ETO was fixed with a cable plate system. The 3D printed porous coated Titanium cable plate was used to bridge the bone defect between proximal and distal femur. A 36 mm inner diameter highly cross-linked polyethylene liner and a 36 mm ceramic head was inserted. Then the joint stability was confirmed after reduction. (Fig. 5)

### 2.5 Postoperative course

**Figure 6:**
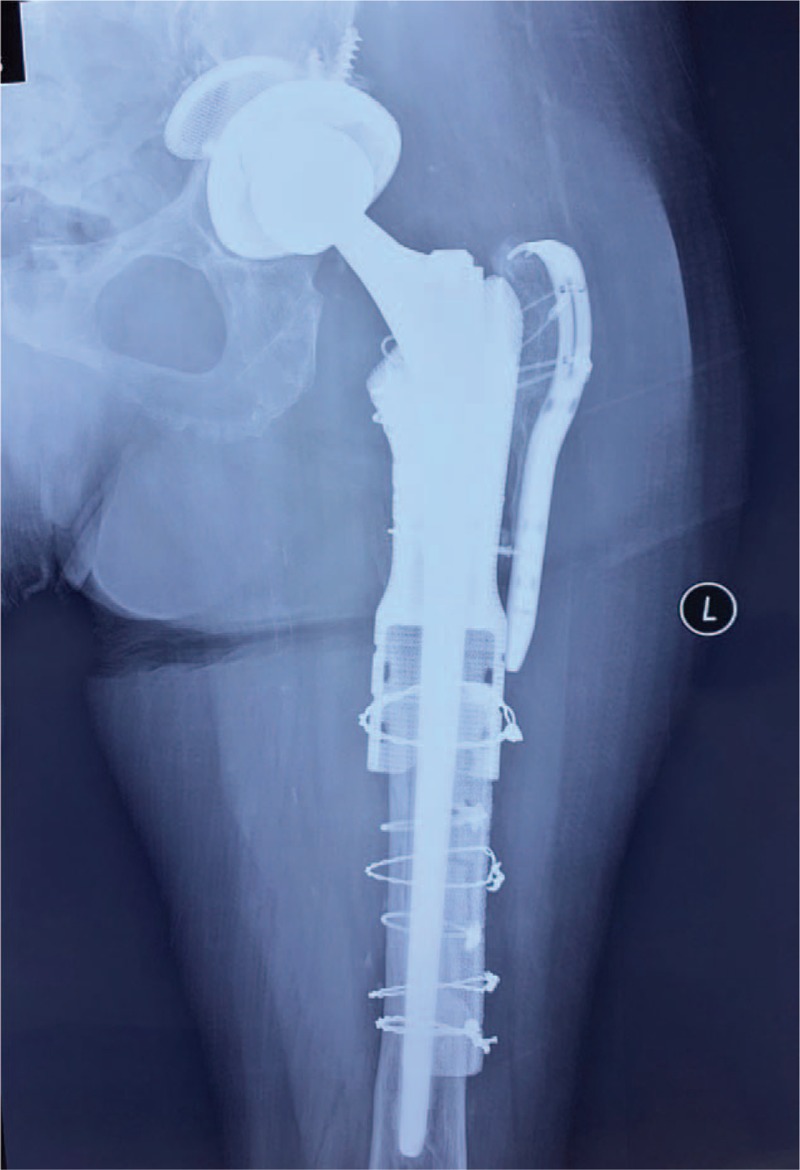
Radiograph at 2 year follow-up showed excellent osteointegration between the interface of sleeve-prosthetic composite and the host bone, with no signs of implant loosening or subsidence.

Postoperatively, non-weight bearing for 6 weeks and then partially-weight bearing for another 6 weeks was applied before full-weight bearing. The patient ambulated independently at 6-month follow up. The total duration of follow-up was 2 years. She was symptom free with a Harris Hip Score of 91 at the 2-year follow-up. Radiographs showed excellent osteointegration between the interface of sleeve-prosthetic composite and the host bone, with no signs of implant loosening or subsidence. (Fig. 6)

## 3 Discussion

Revision total hip arthroplasty for cases with severe bone deficiencies remains a great challenge, especially in cases of significant segmental femoral bone defect, in which, using monoblock or modular revision stem alone is incapable to achieve reliable reconstruction. Megaprosthesis and APCs is often the preferred choice in these cases.^[6]^

Megaprosthesis is originally designed for limb salvage surgery.^[13,14]^ It can be in type of personalized monoblock or modular stem with multiple options of offset, neck length, body length and antevesion. Now its application has extended in revision THA or other non-oncological conditions to manage massive bone defect.^[2,9,15]^ Despite of its less technical comlexity compared with APCs, the dislocation incidence of megaprosthetic replacement is high, contributed to poorly re-established abductor attachment and inadequate soft tissue tension. According to the literature, the incidence of hip dislocation ranges between 6%^[16]^ and 42%.^[17]^ Besides, other complications such as loosening, infection, peri-prosthetic fracture or implant breakage also affect its long term survivorship. According to Parvizi research, the survival rate of proximal femoral replacement was about 72% at 5 years.^[18]^ Its application should be restricted in cases of elder and low activity demanding patients.

APCs can also be used in cases with severe segmental femoral bone defects. An APC is consisted of a bulk proximal femoral allograft and a revision type stem which is cemented into the allograft. The composite can either be cemented or press fit distally into the femoral canal of the host bone. The remaining soft tissue sleeve is attached to the allograft. The allograft is expected to incorporate with the host bone and soft tissue sleeve, to restore bone stock and soft tissue tension. Considering their advantages compared to megaprosthesis, APCs are advocated in younger and active patients. However, reported results of APCs are still unsatisfactory due to complications of non-union, allograft resorption, periprosthetic fracture, infection and dislocation.^[3,4,7–9]^ The survival rate of APCs was about 81% at 8 years^[19]^ and 69% at 10 years.^[3]^

In this case, we attempted to use 3D printed Titanium alloy sleeve-prosthetic composite for reconstruction of segmental bone loss of proximal femur in revision THA. This technique had some advantages over other techniques. First, the personalized 3D printed structure simulated the native anatomy of native femur, helped to restore the proximal femoral anatomy and biomechanics, obviating the need of bone grafting. Second, it maximized the contact surface with host bone, and its porous surface of the composite facilitated bone ingrowth,^[20,21]^ ensured the biological fixation of soft tissue sleeve and implant, thus decreasing the risk of dislocation and loosening. Finally, the 3D printed porous Titanium plate biologically bridged the defect between proximal and distal femur through bone-ingrowth. The restoration of femur continuity and the similar biomechanic properties of the porous Titanium alloy compared to the host bone decreased the stress concentration and the risk of implant failure.

## 4 Conclusion

Although long term result of 3D printed Titanium sleeve-prosthetic composite reconstruction was absent, the good clinical and radiological outcome at 2 years follow up implied its potential role for reconstruction of segmental femoral bone defect in revision THA. More studies are needed to research its potential complications and long term survivorship in future.

## Author contributions

**Conceptualization:** Hui Xu.

**Investigation:** Xingshan Wang, Ji Zhang.

**Methodology:** Xingshan Wang.

**Supervision:** Hui Xu.

**Validation:** Ji Zhang.

**Writing – original draft:** Xingshan Wang.

**Writing – review & editing:** Hui Xu, Ji Zhang.
